# Genetic Dete`rminants of Clozapine-Induced Metabolic and Neurological Side Effects: A Comprehensive Investigation

**DOI:** 10.1192/j.eurpsy.2025.1972

**Published:** 2025-08-26

**Authors:** C. Kalogirou, C. Christou, M. Peyioti, T. Soumala, G. Schoretsanitis

**Affiliations:** 1 University of Patras, Patras, Greece; 2 Marien Hospital, Dusseldorf, Germany; 3 Hospital of Alexandroupolis, Alexandroupolis; 4 University of Ioannina, Ioannina, Greece; 5 Psychiatrische Universitätsklinik Zürich (PUK), Ζürich, Switzerland

## Abstract

**Introduction:**

Clozapine remains one of the most effective antipsychotic medications for treatment-resistant schizophrenia. However, its use is limited by significant metabolic and rare neurological side effects. The variability in patients’ susceptibility to these adverse effects suggests an unidentified genetic predisposition, which is critical to personalizing treatment.

**Objectives:**

This review aims to systematically investigate the genetic variants involved in the development of metabolic and neurological side effects in patients treated with clozapine. Specifically, it seeks to elucidate the pathways participating in the occurrence of these adverse reactions and identify genetic markers for personalized therapeutic approaches.

**Methods:**

A literature search was conducted across multiple databases for studies published in recent years. Data were extracted from cohort studies and case-control studies based on predefined criteria, using keywords such as “clozapine”, “metabolic side effects”, “neurological side effects”, “pharmacogenetics”.

**Results:**

Preliminary results identified that variants in the **FTO** gene, known for its role in adipogenesis and energy homeostasis, were linked to enhanced adiposity and altered leptin signaling, contributing to the metabolic syndrome. Similarly, **APOE** gene was strongly correlated with increased risk of severe weight gain, dyslipidemia, and insulin resistance due to association with impaired lipid transport and metabolism.

In the context of neurological side effects, SNPs within the **DRD2** Taq1A polymorphism, which affects dopamine receptor density and signaling, were associated with a higher incidence of extrapyramidal symptoms and tardive dyskinesia in patients on clozapine. The **COMT** Val158Met polymorphism, known to modulate prefrontal catecholamine degradation, was linked to cognitive impairments and clozapine-induced sedation.

**Next-generation sequencing (NGS)** enlightens rare SNPs, copy number variations (CNVs) and novel variants in genes related to drug metabolism, such as **CYP1A2** and **CYP2D6**, allowing for the identification of complex genetic interactions. Furthermore, patients with high **polygenic risk scores (**PRS) exhibited a markedly increased likelihood of developing severe metabolic and neurological side effects, underscoring the utility of PRS in stratifying patient risk.

**Conclusions:**

In summary, our findings demonstrate that genetic factors are key contributors to the development of clozapine-induced metabolic and neurological side effects. Variations in genes related to drug metabolism, neurotransmitter regulation, and metabolic pathways significantly influence individual susceptibility to these adverse effects. A deeper understanding of pharmacogenetic approaches could offer the potential for more personalized and precise treatment strategies, reducing adverse effects and improving drug tolerance.

**Disclosure of Interest:**

None Declared

